# Supplementary material for: Human Lung Cell Pyroptosis Following Traumatic Brain Injury
Source: Cells. 2019 Jan 18;8(1):69. doi: 10.3390/cells8010069 (PMC6356886; doi:10.3390/cells8010069)
Supplement: Supplementary file 1 [file cells-08-00069-s001.pdf]

**Supplementary Figures**

**Supplementary Figure 1.**

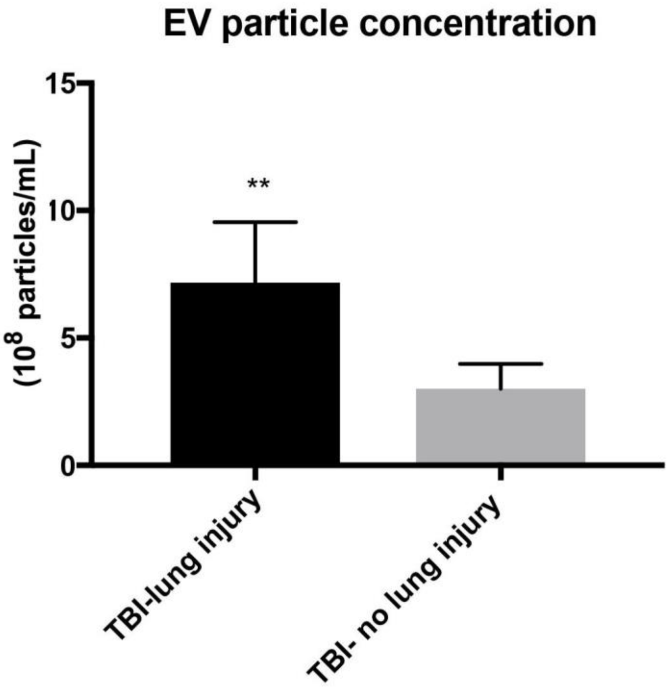


**Figure S1.** Nanosight (NTA) Characterization of serum-derived EVs from TBI patients with and without lung injury. NTA analysis showing significant difference in particle concentration (per mL of PBS) between serum-derived EVs from TBI patients with lung injury (n = 13) and TBI patients with no lung injury (n = 8, ** $p < 0.05$).

**Supplemental Figure 2.**

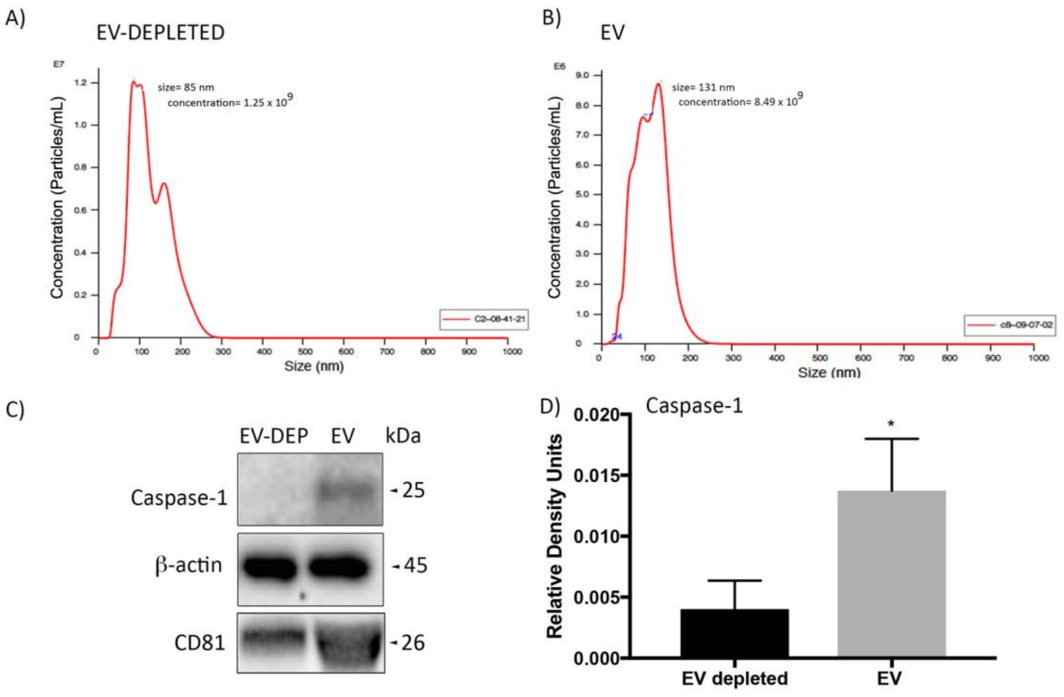


**Figure S2.** EV depleted isolations do not activate the inflammasome in pulmonary endothelial cells. (**A**,**B**) Representative Nanosight analysis shows a 6.9 fold decrease in EVs from $8.49 \times 10^9$ (EVs) to $1.25 \times 10^9$ particles/mL (EV depleted) particles/mL from depleted samples. (**C**,**D**) Representative Western blots (**C**) and quantification (**D**) show that HMVEC-L treated with EV depleted samples have a significantly lower expression of caspase-1, and CD81 after delivery to HMVEC-L ($n = 3$, * $p < 0.05$).
